# Exponential adoption of battery electric cars

**DOI:** 10.1371/journal.pone.0295692

**Published:** 2023-12-11

**Authors:** Felix Jung, Malte Schröder, Marc Timme

**Affiliations:** 1 Chair of Network Dynamics, Center for Advancing Electronics Dresden (cfaed) and Institute of Theoretical Physics, TUD Dresden University of Technology, Dresden, Germany; 2 Lakeside Labs, Klagenfurt, Austria; Università di Pisa, ITALY

## Abstract

The adoption of battery electric vehicles (BEVs) may significantly reduce greenhouse gas emissions caused by road transport. However, there is wide disagreement as to how soon battery electric vehicles will play a major role in overall transportation. Focusing on battery electric passenger cars, we analyze BEV adoption across 17 individual countries, Europe, and the World, and consistently find exponential growth trends. Modeling-based estimates of future adoption given past trends suggest system-wide adoption substantially faster than typical economic analyses have proposed so far. For instance, we estimate the majority of passenger cars in Europe to be electric by about 2031. Within regions, the predicted times of mass adoption are largely insensitive to model details. Despite significant differences in current electric fleet sizes across regions, their growth rates consistently indicate fast doubling times of approximately 15 months, hinting at radical economic and infrastructural consequences in the near future.

## Introduction

Climate change poses a major challenge to humanity in the near to midterm future [1]. It is caused by anthropogenic emissions of greenhouse gases, predominantly carbon dioxide [2–4]. To combat climate change, global carbon dioxide emissions need to be reduced significantly and rapidly. A major contributor to greenhouse gas emissions in general and to those of western countries in particular is the road transport sector [5], to a large degree due to passenger cars propelled by internal combustion engines [6, 7]. In 2019, transportation caused 30% of the greenhouse gas emissions in the United States of America (USA), and 18% in Europe, but only 9% in India, and 7% in China [8]. Over the past decades, however, the transport sector has failed to reduce its carbon footprint [9].

Mitigating carbon emissions from the transport sector requires the transition to carbon-neutral powertrains, and thus the replacement of mineral oil as the underlying source of energy. Today, the two most common options to achieve this goal are to either produce hydrogen by processing biomass or splitting water [10, 11], which is subsequently used to power an electric motor by means of a fuel cell [12], or directly power an electric motor by means of a rechargeable (often lithium-ion-based) battery [13]. For both electrolytic hydrogen and electric power, the carbon-neutrality hinges on the renewable generation of the electric energy.

Several related technologies are also under development. For instance, hydrogen [14] and synthetic fuels, such as *biofuels* or *electrofuels* [15–18], may be used to power internal combustion engines. Moreover, batteries based on elements other than lithium are under active development, most prominently sodium-ion batteries [19, 20].

The technologies required to refuel vehicles based on electric batteries, compressed hydrogen, cryogenic liquid hydrogen, and synthetic fuels have little technological overlap [21, 22] such that several support infrastructure systems would need to be established for multiple powertrain options to coexist. Transitioning from the current largely homogeneous infrastructure supporting vehicles with petrol- and diesel-based internal combustion engines to a heterogeneous infrastructure would likely incur substantially higher operational costs. Such economic reasons may act as additional drivers to single out just one winning technology that dominates the market.

With battery-electric powertrains currently being the most common carbon-neutral option by far, this provokes the question whether they will succeed as the dominant technology, and, if so, when this will happen.

Previous research primarily focuses on factors influencing and incentives driving the transition to electric vehicles. This includes investigating consumer preferences and adoption behavior using surveys, discrete choice models and statistical analysis [23–25], evaluating adoption obstacles, purchase incentives and the effect of policy support [26–29], as well as assessing future technological advances and changes in mobility patterns [30]. Early-adopter electric vehicle uptake has also been studied spatially, taking the effects of charging infrastructure and socioeconomic and demographic characteristics into account [31, 32]. The existing literature on consumers’ perception of EVs, adoption drivers, and adoption barriers has been extensively reviewed in [33–36].

Electric vehicle fleet sizes have primarily been projected for specific regions, employing detailed microscopic and scenario-based modeling to assess the impact of policy changes [37–39]. Rietmann et al. have studied BEC adoption globally by fitting a modified logistic model to past adoption trends [40].

We now strive to answer how soon electric vehicles will dominate passenger car fleets by performing a purely data-driven analysis of the recent worldwide adoption of battery electric cars (BECs), i.e., battery electric light passenger vehicles, create model-based projections of future adoption, and put the results into perspective with regard to the total number of registered passenger cars (PCs).

Our analysis focuses on the major growing BEC markets of Europe and the USA, intentionally excluding developing countries, such as China or India, which, while having large BEC markets, at the same time experience rapid growth of their total passenger car fleets. For example, in recent years, China’s and India’s PC fleets have sustained average annual growth rates of 15% and 9%, respectively [41]. It is unclear in which fashion this total fleet growth will continue into the future, which places the BEC adoption process in these regions outside the scope of this study. Bearing this uncertainty in mind, however, we still provide aggregate worldwide results.

We demonstrate that the observed recent adoption of battery electric cars has happened exponentially. Based on this result, we describe the historic growth trends using three related models: An exponential model, a logistic model and a Bass diffusion model. We then employ these models to estimate a future transition point in the mobility landscape: The point in time at which battery electric vehicles are expected to start dominating the passenger car fleet in a country, i.e., to comprise 50% of the total fleet, *should* the current trend continue.

## Data

Our analysis relies on worldwide historic BEC and PC registration data, now described in further detail.

### Electric vehicle stock

Our data for the number of BECs across various regions have been provided by the International Energy Agency (IEA), an intergovernmental organization, accompanying their *Global EV Outlook 2022* [42]. This dataset provides information on the adoption of electric vehicles and public charging infrastructure in 34 regions worldwide. It includes data from 30 countries (Australia, Belgium, Brazil, Canada, Chile, China, Denmark, Finland, France, Germany, Greece, Iceland, India, Indonesia, Italy, Japan, Korea, Mexico, Netherlands, New Zealand, Norway, Poland, Portugal, South Africa, Spain, Sweden, Switzerland, Thailand, USA, United Kingdom) and four aggregate regions (“Europe”, “Other Europe”, “Rest of the world”, “World”). The dataset contains both year-resolved historical data, ranging from 2011 to 2022, and scenario-based projected future data up to 2031, according to their *Announced Pledges Scenario* (APS) and *Stated Policies Scenario* (STEPS). For several kinds of vehicles (buses, cars, trucks, vans), and the respective powertrain technology (battery electric, plug-in hybrid), sales and stock numbers are provided. The dataset also contains information on charging infrastructure, namely the number of public slow and fast chargers. Finally, for select large regions, values for mineral oil displacement and electricity demand of electric vehicles are provided (China, “Europe”, India, “Rest of the world”, USA, “World”). Not all described data are available for all the countries contained in the dataset.

For our analysis, we extract the year-resolved historic data on the absolute and relative number of battery electric passenger cars per region.

### Passenger car stock

To model the total passenger car fleet, we combine vehicle registration data from several sources.

The International Organization of Motor Vehicle Manufacturers (OICA) [41] provides worldwide vehicle registration data, distinguishing between passenger cars, defined as motor vehicles intended for passenger transport of a maximum capacity of nine persons, and commercial vehicles, such as trucks, coaches, and buses. The dataset contains vehicle stock numbers for the years 2015 and 2020 and 63 countries. For Europe, this includes the categories “EU 27 + EFTA + UK” (Austria, Belgium, Bulgaria, Croatia, Czechia, Denmark, Finland, France, Germany, Greece, Hungary, Ireland, Italy, Netherlands, Norway, Poland, Portugal, Romania, Slovakia, Spain, Sweden, Switzerland, the United Kingdom, and a residual category) and “Russia, Turkey and Other Europe” (Belarus, Russia, Serbia, Turkey, Ukraine, and a residual category), for America, the categories “NAFTA” (Canada, Mexico, USA) and “Central and South America” (Argentina, Brazil, Chile, Colombia, Ecuador, Peru, Venezuela, and a residual category), for Asia, Oceania, and the Middle East, the countries of Australia, China, India, Indonesia, Iran, Iraq, Israel, Japan, Kazakhstan, Malaysia, New Zealand, Pakistan, the Philippines, South Korea, Syria, Taiwan, Thailand, the United Arab Emirates, Vietnam and a residual category, and for Africa, the countries of Algeria, Egypt, Libya, Morocco, Nigeria, South Africa, and a residual category.

For Europe, Eurostat [43], a Directorate-General of the European Commission, provides vehicle registration data. This includes data for countries which are members of the European Union (EU), namely Austria, Belgium, Bulgaria, Croatia, Cyprus, Czechia, Denmark, Estonia, Finland, France, Germany, Greece, Hungary, Ireland, Italy, Latvia, Lithuania, Luxembourg, Malta, Netherlands, Poland, Portugal, Romania, Slovakia, Slovenia, Spain, and Sweden. They furthermore provide data on Liechtenstein, Norway, and Switzerland, which are members of the European Free Trade Association, North Macedonia and Turkey, which are EU membership candidate countries, Kosovo, which is a potential EU membership candidate country, and the United Kingdom, which is a former EU member.

The dataset contains passenger car stock numbers for the respective countries for each year from 2012 to 2019, except for a few missing values. The stock numbers are individually specified for each of the surveyed powertrain technologies: bi-fuel, biodiesel, bioethanol, diesel, diesel (excluding hybrids), electricity, hybrid diesel-electric, plug-in hybrid diesel-electric, hybrid electric-petrol, plug-in hybrid petrol-electric, natural gas, hydrogen and fuel cells, liquefied petroleum gases (LPG), petroleum products, petrol (excluding hybrids), and a residual category. These categories are defined in more detail in [44].

For the USA, the Federal Highway Administration (FHWA) releases annual vehicle registrations data, broken down by state [45]. Registrations are reported individually for passenger cars, buses, trucks, and motorcycles. Additionally, the dataset separates private and commercial registrations on the one hand, and public registrations on the other. The most recent data have been released for 2021. We have extracted data in a common format starting from 2007.

For Iceland, Statistics Iceland, the National Statistical Institute of Iceland, releases data on the number of registered passenger cars [46], from 1950 to most recently 2021 (number at end of year).

An overview of the data sources used for the BEC and PC fleet sizes and the respective years covered is given in Table 1.

**Table 1:** Data sources overview.

Region	Data source PC stock^a^	Data source BEC stock	BEC data range /yr
World	OICA	IEA	[2011, 2022]
Europe	Statistics Iceland, Eurostat, OICA	IEA	[2011, 2022]
Belgium	OICA	IEA	[2011, 2022]
Denmark	OICA	IEA	[2011, 2022]
Finland	OICA	IEA	[2012, 2022]
France	OICA	IEA	[2011, 2022]
Germany	OICA	IEA	[2011, 2022]
Greece	OICA	IEA	[2014, 2022]
Iceland	Statistics Iceland	IEA	[2011, 2022]
Italy	OICA	IEA	[2011, 2022]
Netherlands	OICA	IEA	[2011, 2022]
Norway	OICA	IEA	[2011, 2022]
Poland	OICA	IEA	[2016, 2022]
Portugal	OICA	IEA	[2011, 2022]
Spain	OICA	IEA	[2011, 2022]
Sweden	OICA	IEA	[2011, 2022]
Switzerland	OICA	IEA	[2011, 2022]
United Kingdom	OICA	IEA	[2011, 2022]
Other Europe	Eurostat, OICA	IEA	[2011, 2022]
USA	FHWA	IEA	[2011, 2022]

PC, passenger car; BEC, battery electric car; OICA, International Organization of Motor Vehicle Manufacturers; IEA, International Energy Agency; FHWA, US Federal Highway Administration.

^a^ Total PC stock values $\hat{n}\left(\tilde{t}\right)$ were determined for the year $\tilde{t}=2020\;\text{yr}$, except for those values taken from the Eurostat dataset, where $\tilde{t}=2019\;\text{yr}$.

### Rapid BEC adoption worldwide

**Fig 1:**
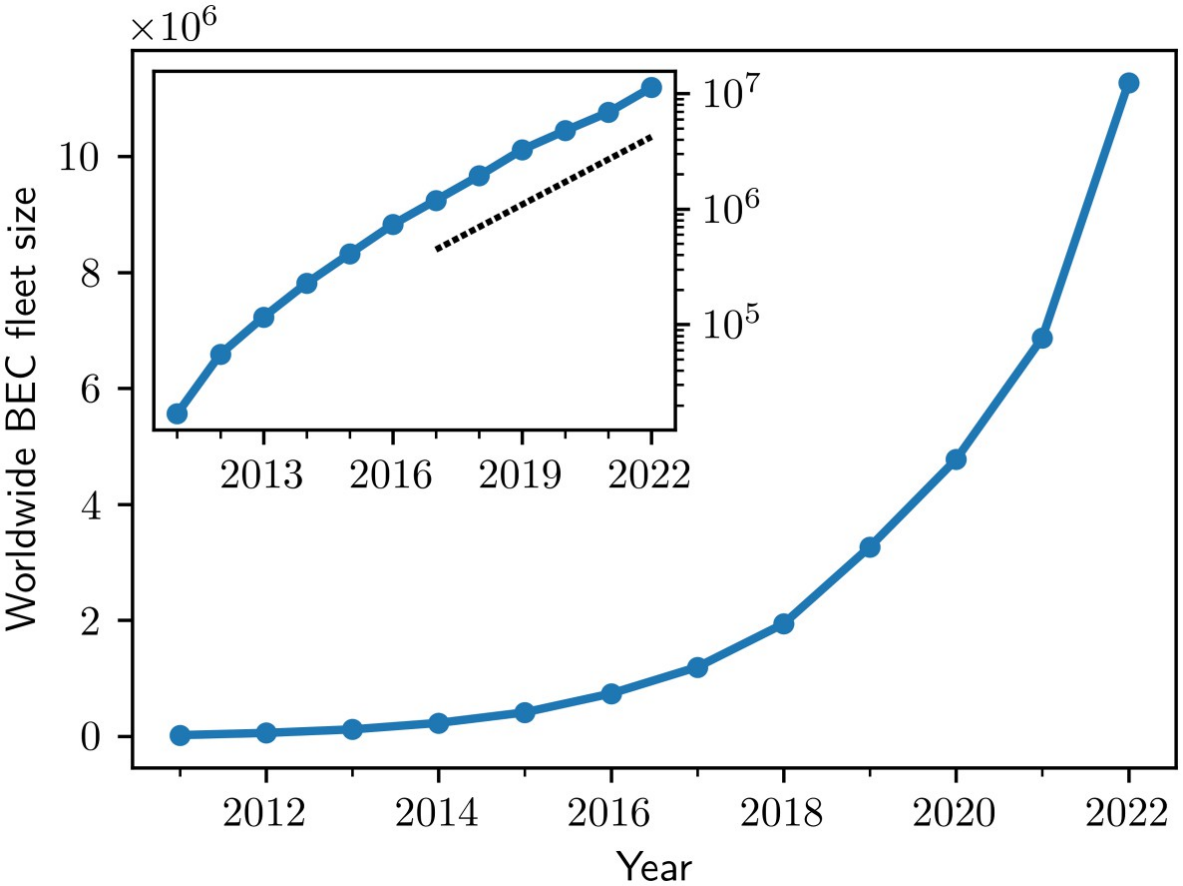
Global exponential growth of BEC adoption. The number of battery electric cars in operation has grown rapidly across the world. The straight-line increase on the logarithmic-linear scale (inset) over two orders of magnitude (black line indicates slope of fit of 2016-2022 data) highlights that the recent adoption has happened exponentially.

Globally, the number of BECs has been growing rapidly, resembling an exponential adoption process, see Fig 1, and reaching a value of approximately 11 million vehicles at the beginning of 2022.

**Fig 2:**
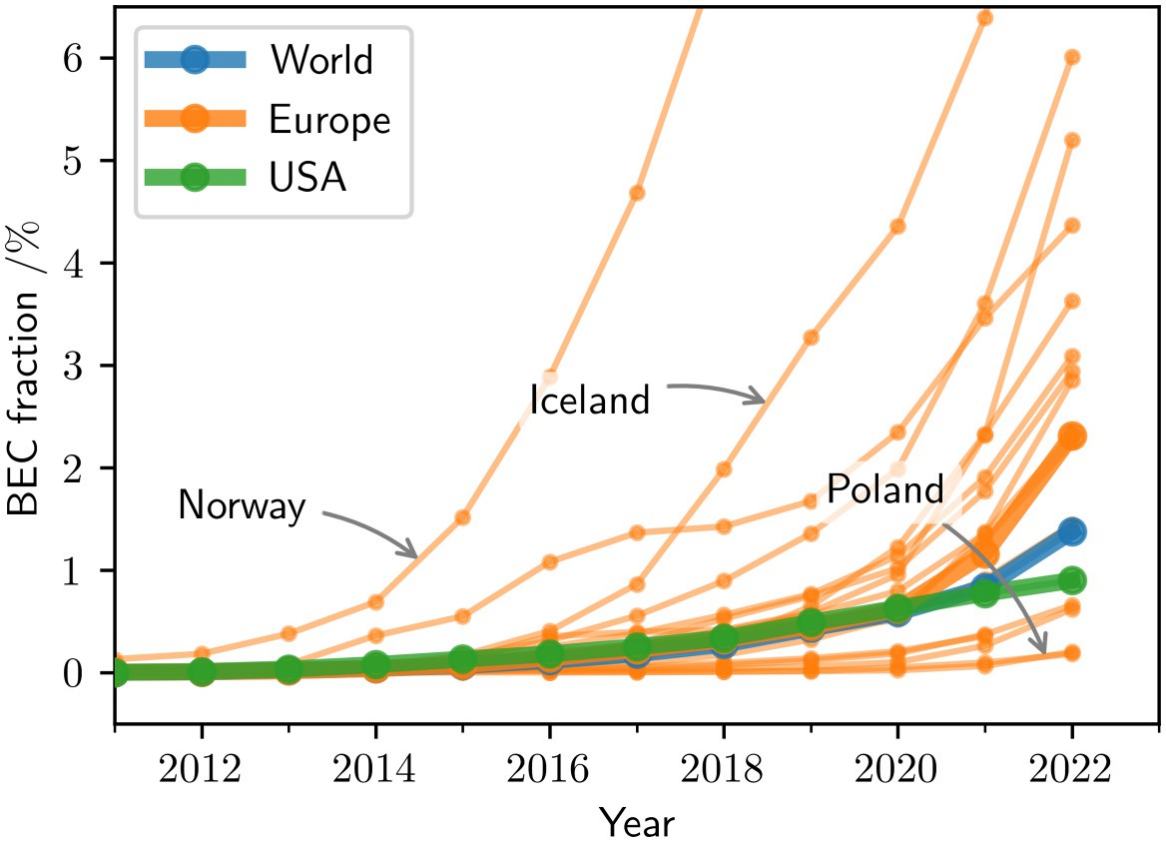
BEC adoption growth across regions. The BEC share in the total PC fleet has grown significantly over the past decade, with individual countries being at very different stages of adoption: In 2022, Norway features a BEC share of 25.3% (out of frame), while Poland is at just under 0.2%. Europe outperforms both the USA and the world average.

The high absolute numbers are reflected in the BEC fraction of the total PC fleet, see Fig 2, reaching approximately 1.4% globally in the beginning of 2022, with Europe having a 2.3% BEC fraction and the USA 0.9%. Comparing the adoption in individual countries reveals significant variation in the current BEC fraction values. In Europe, Norway is leading the way at 25.3% in the beginning of 2022, while at the same time, Poland has the lowest fraction of just under 0.2%, spanning a range of two orders of magnitude.

Whether these very different adoption processes follow a common law and what this means for the dominance of BECs in those countries is the subject of our analysis.

## Methods

We model the historic adoption trends of battery electric cars in 16 individual European countries (Belgium, Denmark, Finland, France, Germany, Greece, Iceland, Italy, Netherlands, Norway, Poland, Portugal, Spain, Sweden, Switzerland, United Kingdom) and the residual category “Other Europe”, containing 17 countries (Austria, Bulgaria, Croatia, Cyprus, Czechia, Estonia, Hungary, Ireland, Latvia, Liechtenstein, Lithuania, Luxembourg, Malta, Romania, Slovakia, Slovenia, Turkey), in the USA, and globally aggregated.

For each of these regions, country or aggregate, we fit three related growth models, an exponential model $n_{\text{E}}^{\left(\text{exp}\right)}$, a logistic model $n_{\text{E}}^{\left(\text{log}\right)}$, and a bass diffusion model $n_{\text{E}}^{\left(\text{bass}\right)}$, to the empiric number of registered BECs ${\hat{n}}_{\text{E}}\left(t\right)$ at times *t* measured in calendar years, ranging from 2011 to 2022. For three countries, data are only available from 2012 (Finland), 2014 (Greece), and 2016 (Poland), cf. Table 1; their fit range is reduced accordingly. The BEC fleet sizes of the aggregate regions “Europe” and “World” are computed by approximating the three countries’ fleet sizes to be zero in the affected years. All three models are described in more detail below.

Similar results might also be obtained with models of battery electric car sales and the corresponding total car sales data. These models would effectively describe the derivatives of the BEC stock, disregarding the sale or scrapping of used BECs—an assumption that might be considered reasonable during the early adoption phase.

**Fig 3:**
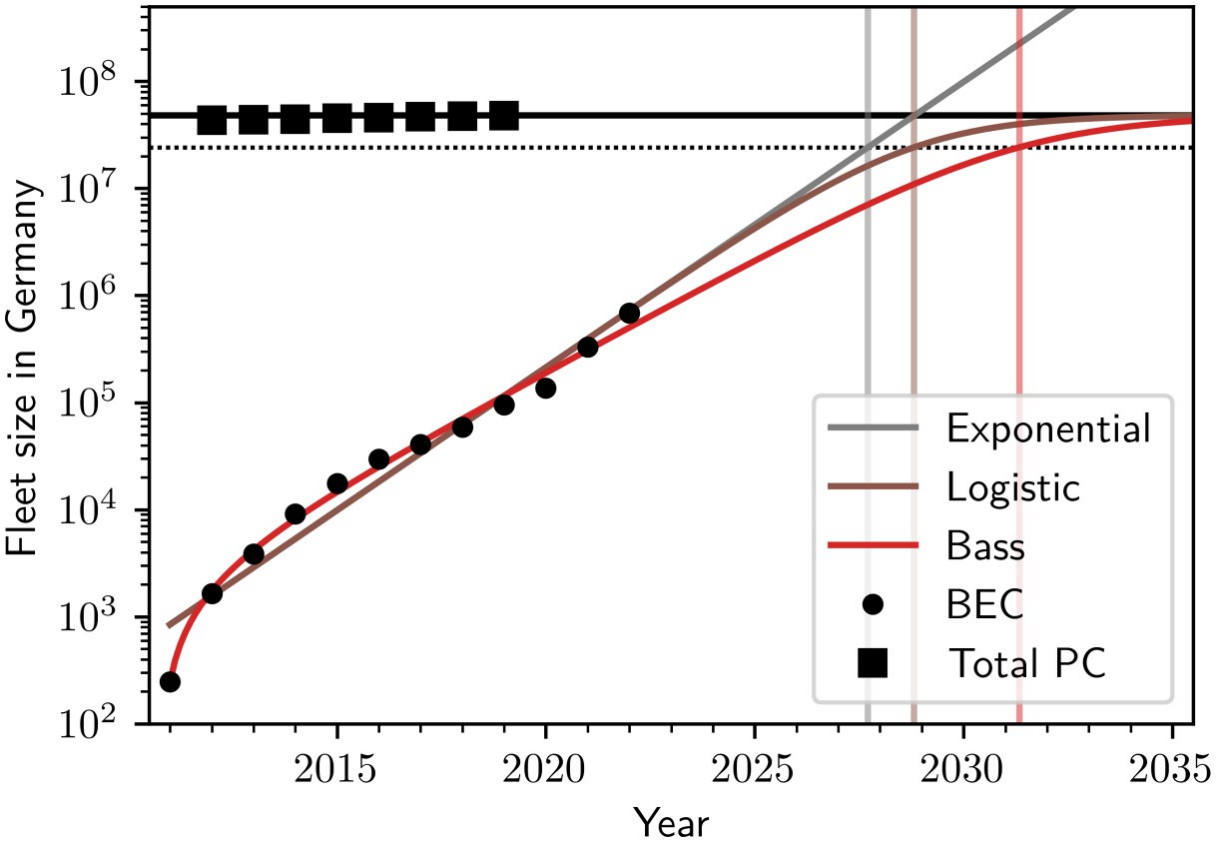
Estimating BEC dominance. Battery electric car fleet sizes may soon dominate the total passenger car fleet, shown here for Germany as an example. The past total passenger car fleet size stayed approximately constant (black horizontal line), with the data points (black squares) taken from the Eurostat dataset. The exponential (gray, Eq (2)), logistic (brown, Eq (5)) and Bass diffusion (red, Eq (8)) models predict the future growth of BEC adoption. The dominance times *t*_1/2_ (gray, brown, red vertical lines) at which half of the passenger car fleet (dotted horizontal line) consists of BECs are estimated to occur between 2027 and 2032, depending on the model.

To estimate the point in time at which BECs start dominating the passenger car fleet, we extrapolate the models into the future. We describe the total passenger car fleet size using a trivial model $n\left(t\right)=\hat{n}\left(\tilde{t}\right)=n$, assuming a constant total number of passenger cars since the year 2020, i.e., we take $\tilde{t}=2020\;\text{yr}$. This choice is motivated by the historic total fleet sizes over the modeling timespan, i.e., since the year 2011, which for the countries considered in this study have been approximately constant (cf. Fig 3), with an unweighted average growth rate across treated regions of 1.5% between 2015 and 2020 [41], and a recent decline of annual sales which may be attributed to the SARS-CoV-2 pandemic and high inflation. The assumption of a constant total PC fleet size likely underestimates the true future value (and may influence the dominance times) in countries with growing total fleet sizes such as China or India, which are therefore not explicitly treated here.

Based on data availability, we gather the total PC fleet size from multiple sources: For the USA, we use the number reported by the FHWA [45], and for Iceland, we use the number reported by Statistics Iceland [46]. We use the numbers reported by the International Organization of Motor Vehicle Manufacturers (OICA) for all other countries contained in the dataset. For the remaining countries (Cyprus, Estonia, Iceland, Kosovo, Latvia, Liechtenstein, Lithuania, Luxembourg, Malta, North Macedonia, and Slovenia), we resort to the latest number reported by Eurostat [43] for 2019 (for those countries, $\tilde{t}=2019\;\text{yr}$), cf. Table 1.

For the aggregate region “Other Europe”, we sum the values for the PC fleet size from the sources described above (summing over Cyprus, Estonia, Iceland, Kosovo, Latvia, Liechtenstein, Lithuania, Luxembourg, Malta, North Macedonia, and Slovenia).

Similarly, for the aggregate region “Europe”, we sum over the individual countries (Belgium, Denmark, Finland, France, Germany, Greece, Iceland, Italy, Netherlands, Norway, Poland, Portugal, Spain, Sweden, Switzerland, United Kingdom) and “Other Europe”.

For the aggregate region “World”, we directly use the aggregate global value for the year 2020 provided by the OICA.

Using the models for the fleet sizes of BECs *n*_E_(*t*) and PCs *n*(*t*) = *n*, we determine the points in time *t*_1/2_ at which BECs are expected to start dominating the PC fleet, i.e. *n*_E_(*t*) = *n*/2, according to the three fitted models, using the construction shown in Fig 3 for the example of Germany, with the historic PC fleet sizes taken from the Eurostat dataset.

### Exponential model

A growth process of a quantity $n_{\text{E}}^{\left(\text{exp}\right)}\left(t\right)$, whose growth rate (i.e., the change of $n_{\text{E}}^{\left(\text{exp}\right)}$ over a time *t*) is proportional to the quantity itself, is called *exponential*. In its simplest form, this dynamics is formalized by the differential equation

$$\begin{array}{c} \frac{\text{d}n_{\text{E}}^{\left(\text{exp}\right)}(t)}{\text{d}t}=an_{\text{E}}^{\left(\text{exp}\right)}(t) \end{array}$$ (1)

with a single parameter denoting the growth rate *a* > 0.

Its solution motivates our *exponential model* for the number of BECs,

$$\begin{array}{c} n_{\text{E}}^{\left(\text{exp}\right)}\left(t\right)=\text{exp}(a(t-t_{0}))\;a,t_{0},t>0, \end{array}$$ (2)

with a temporal offset *t*_0_.

Exponential models have previously been employed in modeling innovation diffusion at the beginning of an adoption process, modeling the adoption as a pure imitation process [47–50].

Assuming multiplicative measurement errors, i.e., a constant percentage error, we perform a nonlinear least squares regression of the logarithmized version

$$\begin{array}{c} \text{log}\;n_{\text{E}}^{\left(\text{exp}\right)}\left(t\right)=a\left(t-t_{0}\right) \end{array}$$ (3)

of the model to estimate the two parameters *a* and *t*_0_.

### Logistic model

While the exponential model described above is well suited for modeling initial adoption, it lacks the idea of ultimate saturation, mathematically suggesting indefinite growth. The logistic model introduces such a saturation limit by making the growth rate of a quantity $n_{\text{E}}^{\left(\text{log}\right)}$ additionally proportional to the difference between the quantity and that limit. In its simplest form, the process is characterized by modifying the differential Eq (1) to become

$$\frac{\text{d}n_{\text{E}}^{\left(\text{log}\right)}(t)}{\text{d}t}=an_{\text{E}}^{\left(\text{log}\right)}(t)(1-n_{\text{E}}^{\left(\text{log}\right)}(t)).$$ (4)

Its solution motivates our *logistic model* for the number of BECs, given by

$$\begin{array}{c} n_{\text{E}}^{\left(\text{log}\right)}\left(t\right)=\frac{L}{1+\text{exp}(-a(t-t_{0}))}\;L,a,t_{0},t>0, \end{array}$$ (5)

with three model parameters: an initial growth rate *a*, a time offset *t*_0_ and the saturation limit *L*.

The logistic model is an established tool to describe technology adoption, having been employed in a number of studies [51–55].

Like for the exponential model, we assume multiplicative measurement errors and perform the parameter optimization of the parameters *a* and *t*_0_ on the logarithmized form

$$\begin{array}{c} \text{log}\;n_{\text{E}}^{\left(\text{log}\right)}\left(t\right)=\text{log}(\frac{L}{1+\text{exp}(-a(t-t_{0}))}) \end{array}$$ (6)

by non-linear least squares regression. We keep the saturation limit *L* fixed to our constant model of the total PC fleet size, i.e., *L* = *n*.

### Bass diffusion model

Explicitly modeling consumer behavior during the adoption of new products, Bass [56] derived the *Bass diffusion model*, treating both initial and replacement purchases, and classifying customers as *innovators* or *imitators*. The Bass model is widely used to describe innovation diffusion [57–61].

It describes the growth of the number of BECs $n_{\text{E}}^{\left(\text{bass}\right)}$ by the differential equation

$$\begin{array}{c} \frac{\text{d}n_{\text{E}}^{\left(\text{bass}\right)}(t)}{\text{d}t}=p(L-n_{\text{E}}^{\left(\text{bass}\right)}(t))+\frac{q}{L}n_{\text{E}}^{\left(\text{bass}\right)}(t)(L-n_{\text{E}}^{\left(\text{bass}\right)}(t))\;L,q,p>0, \end{array}$$ (7)

with three parameters: the *coefficient of innovation p*, the *coefficient of imitation q*, and the saturation limit *L*. The first term, $p(L-n_{\text{E}}^{\left(\text{bass}\right)}\left(t\right))$, describes adoption due to the *innovators*, the second term, $\frac{q}{L}n_{\text{E}}^{\left(\text{bass}\right)}\left(t\right)\left(L-n_{\text{E}}^{\left(\text{bass}\right)}\left(t\right)\right)$, represents adoption due to the *imitators* [62].

Solving the equation with $n_{\text{E}}^{\left(\text{bass}\right)}\left(0\right)=0$, we use it to model the number of BECs in the following form:

$$\begin{array}{c} n_{\text{E}}^{\left(\text{bass}\right)}\left(t\right)=L\frac{1-\text{exp}(-\left(p+q\right)\left(t-t_{0}\right))}{1+qp^{-1}\;\text{exp}(-\left(p+q\right)\left(t-t_{0}\right))}\;L,q,p,t_{0},t>0, \end{array}$$ (8)

with a total of four model parameters: *p* and *q*, a temporal offset *t*_0_ and the saturation limit *L*.

As before, we assume multiplicative measurement errors and perform the parameter optimization of the parameters *p*, *q* and *t*_0_ on the logarithmized form

$$\begin{array}{c} \text{log}\;n_{\text{E}}^{\left(\text{bass}\right)}\left(t\right)=\text{log}(L\frac{1-\text{exp}(-\left(p+q\right)\left(t-t_{0}\right))}{1+qp^{-1}\;\text{exp}\left(-\left(p+q\right)\left(t-t_{0}\right)\right)}) \end{array}$$ (9)

using non-linear least squares regression. Like for the logistic model, we keep the saturation limit *L* fixed to our constant model of the total PC fleet size, i.e., *L* = *n*.

## Results

We report two types of results, one on extracting past trends of BEC adoption, as provided by fitting the three classes of models to past data, and the second on estimating future BEC adoption, should the past trends continue according to these models. The numerical results of our model fitting procedure are listed in Table 2, which for all countries provides the optimal parameter estimates (“Fit” columns), as well as the constant model parameters (“Fixed” columns), and the respective values of $R_{\text{adj}}^{2}$ and the root-mean-square deviation RMSD (“Properties” columns).

**Table 2:** BEC growth model parameters and fit properties.

Model	Exponential				Logistic					Bass					
Parameter type	Fit		Properties		Fit		Fixed	Properties		Fit			Fixed	Properties	
Parameter	*a*/yr^−1^	*t*_0_/yr	$R_{\text{adj}}^{2}$	RMSD	*a*/yr^−1^	*t*_0_/yr	*L*	$R_{\text{adj}}^{2}$	RMSD	*p*/yr^−1^	*q*/yr^−1^	*t*_0_/yr	*L*	$R_{\text{adj}}^{2}$	RMSD
World	0.56	1992.30	0.79	1372054	0.56	2029.74	1180677016	0.80	1356589	2.16 × 10^−5^	0.47	2010.44	1180677016	0.99	319369
Europe	0.51	1992.69	0.98	121609	0.51	2030.84	307157761	0.98	119542	2.20 × 10^−5^	0.44	2010.16	307157761	0.98	107877
Belgium	0.53	2001.29	0.94	3463	0.53	2030.47	5827195	0.94	3413	3.06 × 10^−5^	0.41	2010.67	5827195	0.97	2375
Denmark	0.51	2000.09	0.92	4557	0.51	2029.15	2720273	0.92	4560	1.23 × 10^−4^	0.34	2010.83	2720273	0.80	6993
Finland	0.57	2004.93	0.90	1908	0.57	2030.95	2748448	0.89	1923	1.00 × 10^−7^	0.57	2003.64	2748448	0.88	1931
France	0.55	1997.35	0.54	80141	0.55	2029.32	38458212	0.55	79591	7.02 × 10^−5^	0.37	2010.89	38458212	0.98	14425
Germany	0.61	2000.02	0.97	31237	0.62	2028.81	48248584	0.97	30958	2.16 × 10^−5^	0.49	2010.78	48248584	0.88	56284
Greece	0.70	2010.58	0.95	201	0.70	2032.80	5315875	0.95	201	3.72 × 10^−6^	0.47	2013.85	5315875	0.61	496
Iceland	0.64	2006.83	0.52	1930	0.65	2026.24	269825	0.56	1859	2.31 × 10^−5^	0.60	2009.87	269825	0.78	1228
Italy	0.45	1997.33	0.75	15569	0.45	2036.02	39717874	0.75	15589	1.00 × 10^−7^	0.45	2002.08	39717874	0.71	15664
Netherlands	0.58	2000.19	0.96	14398	0.58	2027.94	9049959	0.96	13561	3.31 × 10^−5^	0.51	2010.21	9049959	0.96	13957
Norway	0.48	1993.83	0.77	60735	0.50	2024.58	2794457	0.84	50223	5.07 × 10^−4^	0.43	2009.77	2794457	0.97	22185
Poland	0.62	2006.17	1.00	125	0.62	2033.61	25113862	1.00	126	1.25 × 10^−7^	0.62	2008.75	25113862	1.00	135
Portugal	0.38	1994.81	0.88	3500	0.38	2035.99	5300000	0.88	3519	1.00 × 10^−7^	0.38	1995.72	5300000	0.86	3525
Spain	0.54	2001.01	0.90	6008	0.54	2032.40	25169158	0.90	5983	9.29 × 10^−6^	0.42	2010.69	25169158	0.99	2105
Sweden	0.74	2005.55	0.30	23784	0.74	2026.40	4944067	0.32	23364	3.04 × 10^−5^	0.53	2010.97	4944067	0.98	4031
Switzerland	0.53	1999.98	0.81	9921	0.53	2028.95	4728444	0.82	9736	5.86 × 10^−5^	0.43	2010.50	4728444	0.99	1606
United Kingdom	0.47	1995.13	0.94	24991	0.47	2032.04	36454665	0.94	25324	2.67 × 10^−6^	0.46	2006.24	36454665	0.92	27104
Other Europe	0.54	1999.46	0.93	11486	0.54	2032.27	50296863	0.93	11431	1.06 × 10^−5^	0.42	2010.70	50296863	0.97	6950
USA	0.50	1992.48	0.18	363801	0.50	2029.39	105135300	0.20	361173	1.02 × 10^−4^	0.36	2010.69	105135300	0.90	118900

$R_{\text{adj}}^{2}$ , adjusted R^2^; RMSD, root-mean-square deviation; Fit, parameters determined by the fitting procedure; Fixed, parameters set to constant values; Properties, goodness-of-fit properties of the models.

Past trends confirm current exponential BEC adoption (cf. Fig 1). The exponential model matches the current BEC adoption in most regions with high accuracy (Table 2) despite the large differences in the current fleet penetration of BECs across countries (cf. Fig 2). Importantly, it suggests current growth rates similar to the logistic model, which includes a saturation limit. These results highlight that the current growth is close to purely exponential and saturation effects are still negligible.

**Fig 4:**
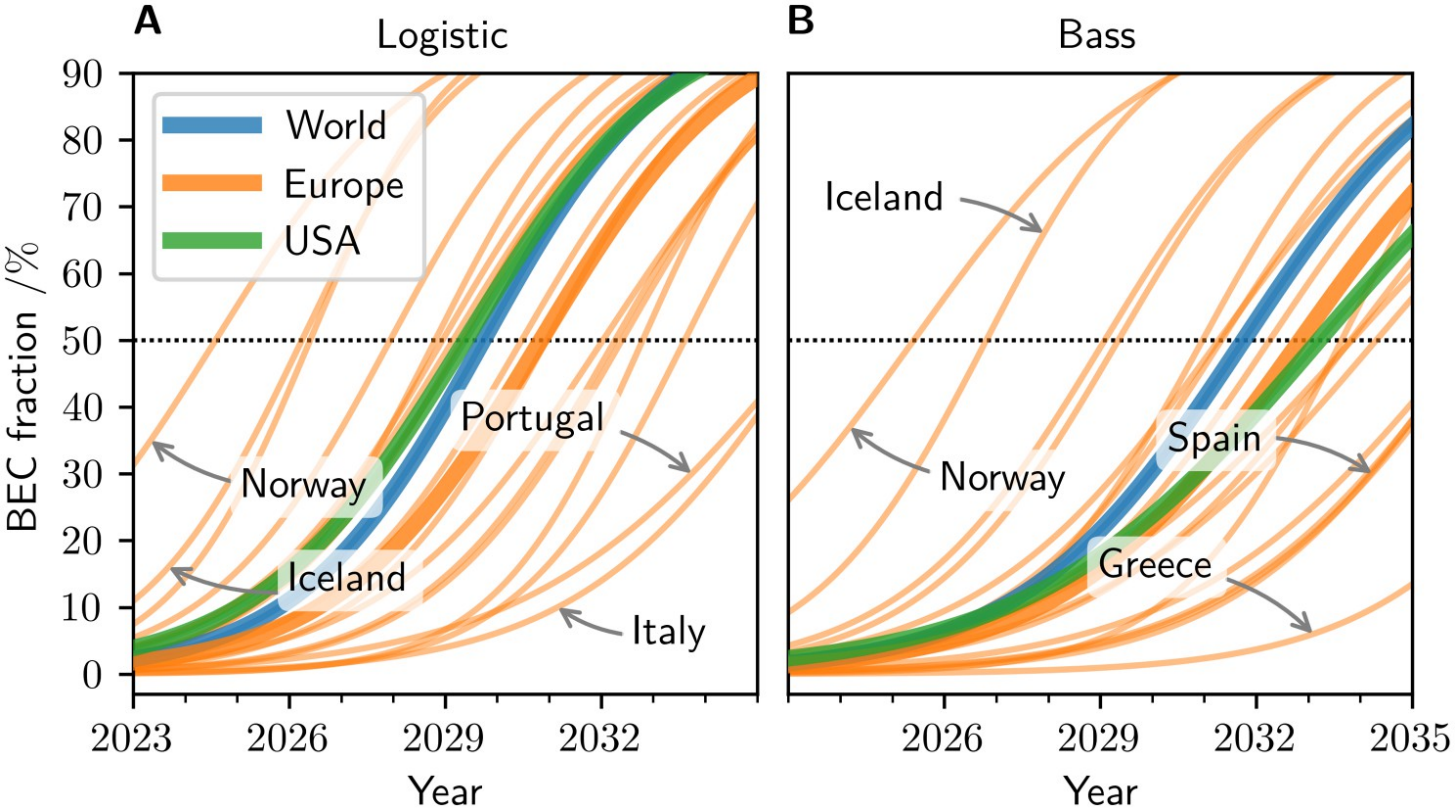
Model-estimated BEC adoption trajectories. The speed of BEC adoption varies strongly across countries (thin orange lines denote individual European countries) in both the logistic model (A) and the Bass model (B). Norway, Sweden, and the Netherlands exhibit the fastest adoption, Spain, Portugal, Italy, and Greece the slowest. Overall, the models predict rapid dominance of BECs (more than 50% of the PC fleet, horizontal gray line) in Europe, the USA and globally shortly after 2030.

When predicting the possible adoption pathways farther into the future, saturation effects play a more significant role. Fig 4 shows the resulting adoption trajectories estimated by the logistic and Bass models. The Bass model consistently provides more conservative trajectories than the logistic model. Within the models’ respective families of adoption curves, the large aggregate regions (World, Europe, USA) exhibit similar adoption dynamics, while the individual European countries differ significantly with respect to the current stage of BEC adoption. While some countries, such as Norway and Iceland, are estimated to undergo a transition to BEC dominance in the near future, others, such as Italy and Portugal, are estimated to reach the BEC dominance about a decade later. These differences apply similarly for both the logistic and Bass models.

**Fig 5:**
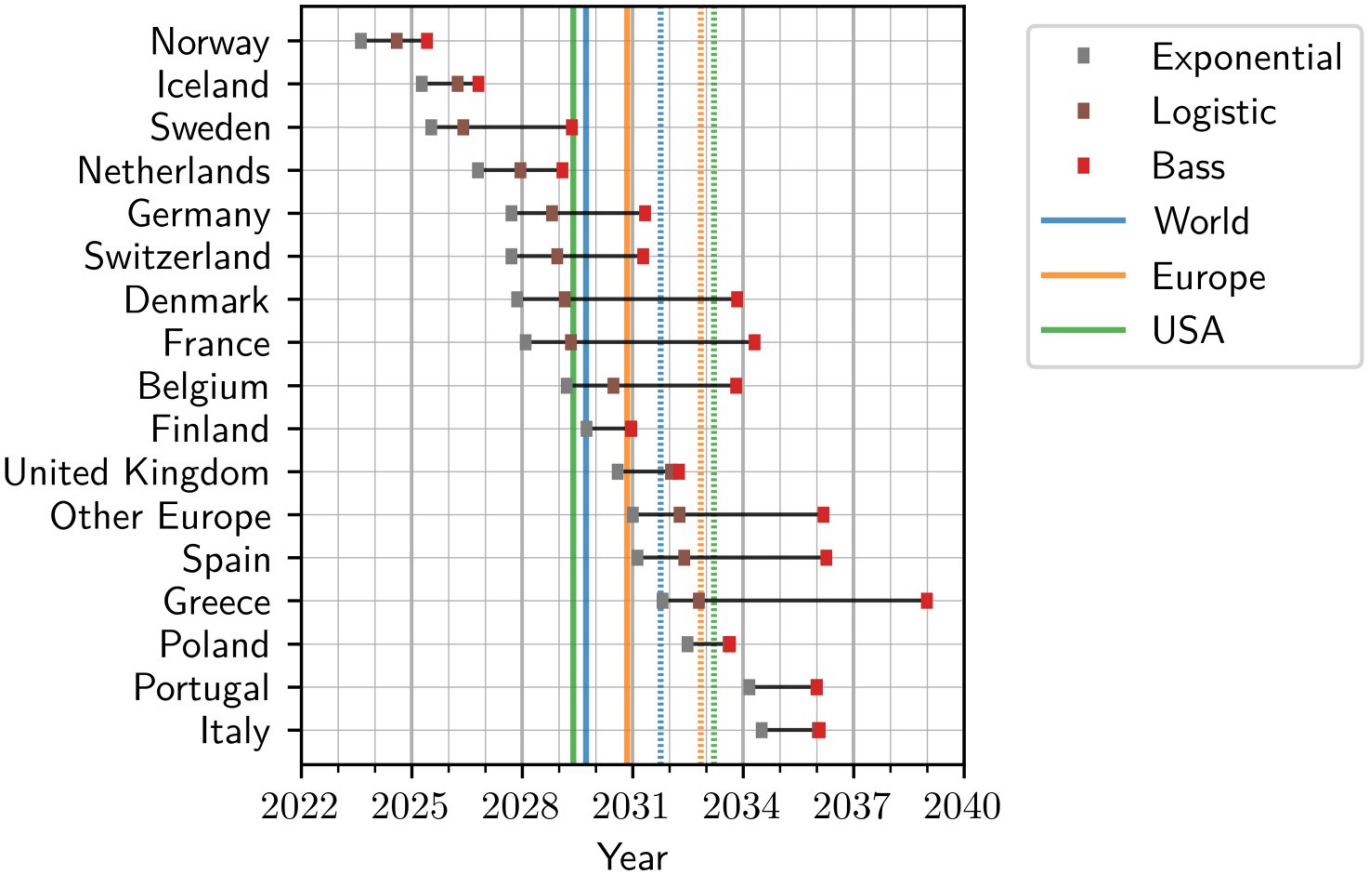
BEC dominance time estimates. Battery electric cars start to dominate the passenger car fleet in European countries between 2024 and 2039, according to our models. The ranking of the estimated dominance times of the countries is consistent across the three models, with the exponential model giving the earliest time, the logistic model an intermediate time, and the Bass diffusion model yielding the latest time. The dominance time estimates for the USA and the aggregate regions “Europe” and “World” are indicated by vertical lines, according to the logistic (solid lines) and Bass diffusion (dotted lines) models.

The spread in the stage of individual regions within their transition to BEC dominance is reflected in the estimated dominance times *t*_1/2_ visualized in Fig 5, computed for each of the three BEC models (exponential, logistic, Bass diffusion). The corresponding numeric values are listed in Table 3. For each region, the three models predict the transition to BEC dominance in a consistent rank order, with the exponential models yielding the earliest estimates, the Bass diffusion models the most conservative estimates and the logistic models intermediate ones, $t_{1/2}^{\left(\text{exp}\right)}\leq t_{1/2}^{\left(\text{log}\right)}\leq t_{1/2}^{\left(\text{bass}\right)}$. Despite the lack of a saturation limit in the former, the estimates of the exponential models and the logistic models are consistently close to each other, with differences of the order of one year. This again highlights the approximate validity of assuming exponential growth up to the point of dominance, as a lowest-order estimate.

**Table 3:** BEC dominance time estimates.

Region	Exponential		Logistic		Bass	
	$t_{1/2}^{\left(\text{exp}\right)}/\text{yr}$	CI-95 /yr	$t_{1/2}^{\left(\text{log}\right)}/\text{yr}$	CI-95 /yr	$t_{1/2}^{\left(\text{bass}\right)}/\text{yr}$	CI-95 /yr
World	2029	[2027, 2030]	2030	[2028, 2031]	2032	[2031, 2033]
Europe	2030	[2028, 2031]	2031	[2029, 2032]	2033	[2031, 2034]
Belgium	2029	[2027, 2032]	2030	[2028, 2033]	2034	[2032, 2035]
Denmark	2028	[2025, 2031]	2029	[2026, 2032]	2034	[2030, 2037]
Finland	2030	[2029, 2031]	2031	[2030, 2032]	2031	– ^a^
France	2028	[2025, 2032]	2029	[2026, 2032]	2034	[2032, 2036]
Germany	2028	[2026, 2030]	2029	[2026, 2031]	2031	[2029, 2033]
Greece	2032	[2029, 2037]	2033	[2029, 2036]	2039	[2033, 2043]
Iceland	2025	[2024, 2027]	2026	[2024, 2028]	2027	[2025, 2029]
Italy	2034	[2032, 2037]	2036	[2033, 2038]	2036	– ^a^
Netherlands	2027	[2025, 2028]	2028	[2026, 2029]	2029	[2027, 2031]
Norway	2024	[2022, 2025]	2025	[2023, 2026]	2025	[2024, 2027]
Poland	2032	[2032, 2033]	2034	[2033, 2035]	2034	– ^a^
Portugal	2034	[2031, 2038]	2036	[2032, 2039]	2036	– ^a^
Spain	2031	[2029, 2034]	2032	[2030, 2035]	2036	[2035, 2038]
Sweden	2026	[2023, 2029]	2026	[2024, 2029]	2029	[2028, 2030]
Switzerland	2028	[2026, 2030]	2029	[2027, 2031]	2031	[2029, 2033]
United Kingdom	2031	[2029, 2032]	2032	[2031, 2033]	2032	– ^a^
Other Europe	2031	[2029, 2034]	2032	[2030, 2034]	2036	[2035, 2038]
USA	2028	[2026, 2031]	2029	[2027, 2032]	2033	[2031, 2036]

exp, exponential model; log, logistic model; bass, Bass diffusion model; *t*_1/2_, time at which the respective model predicts half of the passenger car fleet to be battery electric; CI-95, rounded 95% confidence interval for *t*_1/2_.

^a^ For these regions, the Bass model regression procedure returned no finite error estimate due to the small number of data points.

Some countries (like France and the USA) exhibit large differences in the estimates of dominance times between the three models. These countries show a fast initial increase of BEC fleet size in 2011 to 2013 before settling into a slower but still exponential adoption. Consequently, the exponential and logistic model may slightly overestimate the adoption rate and therefore underestimate dominance times for these countries, reflected also in the lower quality of the fits (see Table 2). In comparison, the Bass model more accurately captures this decrease in the adoption rate, thus predicting higher dominance times.

**Fig 6:**
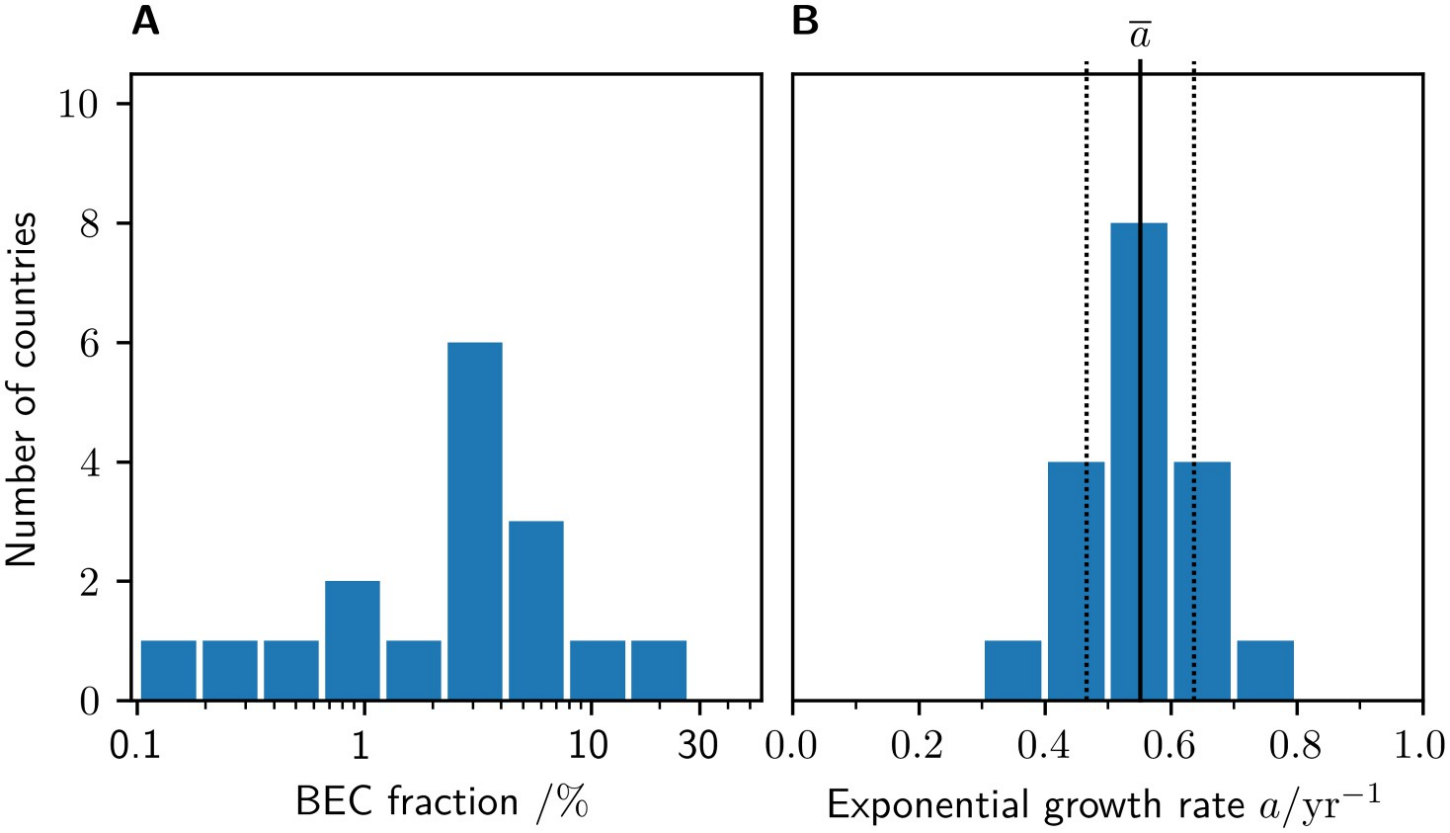
Stage and growth of BEC adoption across countries. A: The current (2022) stage of BEC adoption strongly varies across individual countries, ranging from 0.18% in Poland to 25.3% in Norway (note the logarithmically-scaled abscissa). B: All countries exhibit similarly large growth rates near the average of $\bar{a}=0.55/\text{yr}$ (solid vertical line), yielding a standard deviation of *σ*_*a*_ = 0.09/yr (dotted vertical lines).

In spite of the individual countries representing distinctly different stages of the transition to BEC dominance (Fig 6A), their exponential growth rates *a* are remarkably similar, with an arithmetic mean of $\bar{a}=0.55/\text{yr}$ and a standard deviation of just *σ*_*a*_ = 0.09/yr (Fig 6B). This finding amounts to a doubling time of typically much less than two years (1.26 years on average) in the early phase of adoption.

The dominance time estimates of the large aggregate regions (World, Europe, USA) according to the logistic and Bass model (vertical lines in Fig 5) are close to each other, spanning a range of only approximately 1.5 years for each model (2029 to 2031 in the logistic, 2031 to 2033 in the Bass model). The dominance time estimate for the World is to be taken with a grain of salt, though, as the assumption of a constant PC fleet size *n*(*t*) is unlikely to hold, in particular in developing countries with passenger car fleet sizes that are still small.

## Discussion

Our analysis demonstrates that battery electric cars have been adopted exponentially across the globe for the past decade. Despite the differences in current BEC fleet sizes, both the qualitative exponential trends and the growth rates are highly consistent among countries and regions. The results suggest worldwide BEC dominance less than a decade into the future, potentially much earlier. Exact numerical values of future BEC numbers are impossible to capture in any modeling setting and will depend on several economic, societal, political, and technological factors (see below for further discussion). Such basic modeling is specifically neglecting (unpredictable) future incentives or disincentives, and technological advances, not just specifically in the realm of battery-electric powertrains but also generally for cars and other modes of transport.

The modeling of future adoption *numbers* reported above may thus be seen as extending past trends and suggesting future numbers by order of magnitude. All three models strongly simplify the adoption dynamics. Of the three models compared here, the Bass model incorporates more effects than the simpler exponential and logistic model and may be the most accurate in predicting future BEC fleet size growth, consistently achieving the best fits to the data. However, the expected *time* of BEC dominance is largely insensitive across models as long as a strongly superlinear adoption trend prevails. For instance, for exponential growth patterns in fleet size, the time of adoption at a certain BEC fraction, say 50%, only logarithmically varies with the predicted number of BECs. For the logistic and Bass models, the dependence of dominance times on total adoption numbers is also approximately logarithmic and such that time point estimates within the model classes are similarly insensitive.

Our projected estimates focus primarily on the trends in developed regions with approximately stagnant total PC fleet sizes, which makes it possible to model the total PC fleet size as constant in time. Due to the aforementioned logarithmic behavior of the dominance times, estimates are similarly robust against small growth changes of the total PC stock over time. This condition is not satisfied, however, for developing countries such as China and India, where the total PC stock currently grows significantly [41]. The combination of strong PC market growth and a concurrent, accelerating transition to BECs requires more detailed modeling of both the total PC stock and the BEC stock for these regions.

On the flip side, as the PC markets in developed countries lack the clear upward trend, manufacturers may struggle to accommodate the demand for new cars at the high rates implied by the estimated adoption trajectories short of reaching dominance, delaying the transition. A fixed finite market size would essentially limit the adoption to linear growth at a fixed rate once all new cars are battery electric. This effect is most pronounced for the exponential model, which does not feature saturation. For the Bass diffusion model, depending on the region, the maximum predicted yearly BEC market volume relative to the historic PC peak market volume ranges from 97% to 222%, Poland being an outlier at 616%, with peak sales data taken from [63–80] (data from 2005 for the USA, and from 1995 for the rest). Belgium and Denmark experience maximum predicted relative market volumes of strictly less than 100% and are thus not expected to exhibit finite market effects. It is important to note that the PC market volume strongly fluctuates: on average, the peak yearly sales volumes surpass the corresponding minimum yearly sales volumes in the same time frame by a factor of 300%. It may thus be reasonable to assume that markets will be able to accommodate the large projected demands. Regardless, in the context of our modeling, these high yearly sales volumes are expected only shortly before the dominance time. This suggests that, as BEC fraction doubling times considering the exponential model are on the order of 15 months, a fixed finite market size would only delay the dominance transition by approximately one year across most countries, leaving the overall prediction of BEC dominance within the next decade unchanged.

As stated, while the currently observed absolute fleet sizes and the BEC shares in the total passenger car fleet differ significantly among regions, all of them exhibit exponential growth patterns at similar exponential growth rates *a*. Country-level analysis suggests the same trends for all countries with substantial numbers of BECs to start with. These results indicate a global transition towards cars with battery-electric powertrains, with individual countries and regions currently being at different stages of this transition.

In countries worldwide, governments are pushing for a phase-out of fossil-fuel-powered vehicles [81] to achieve greenhouse gas emission reduction targets. For the European Union, aiming for carbon neutrality in 2050, the European Parliament and Council have agreed to ensure that “all new cars and vans registered in Europe will be zero-emission by 2035” [82]. This is binding for the 27 EU member states and, pending adoption by the EEA (European Economic Area) Joint Committee, for some or all EEA European Free Trade Association (EFTA) states [83]. For the USA, no federal phase-in target has been enacted except for government-owned vehicles, but individual states (California, Massachusetts, New York, Oregon, Vermont, and Washington) have committed to restrict sales of new passenger cars to battery electric, fuel cell electric, and plugin-hybrid electric vehicles [83].

While these government commitments will partially enforce the transition to carbon-neutral powertrains, different options for homogeneous or heterogeneous technology adoption are still discussed to date. These include electric powertrains using batteries or hydrogen fuel cells as means of energy storage, as well as internal combustion engines using electrofuels. Additionally, if and how fast these technologies will make up a significant part of the fleet and whether the transition will be driven by consumer behavior or by government restrictions is subject to debate. This debate is reflected in research and industry forecasts [84, 85], along with those of market analysts [86].

In contrast to the results of our data-driven modeling reported above, earlier work by Statharas et al. estimates the total EV share (both battery electric and plugin-hybrid electric vehicles) in the EU at only up to 21% by 2030 through simulations employing agent-based transport modeling [37]. Rietmann et al. predict 30% of passenger cars to be EVs by 2032, with much higher numbers for several “Fast EV penetration” countries (Austria, Denmark, Portugal, UK, Germany, Netherlands) [40]. Using a complex framework which models the entire energy sector, Kapustin et al. predict only 12–28% of the global fleet to be electric in 2040, depending on the scenario [38].

Current longer term projections of business intelligence firms and market analysts polarize heavily. The management consulting firm McKinsey & Company predicts rapid BEV adoption and market dominance near the turn of the decade [87], the strategic market research provider BloombergNEF similarly expects 50% of all cars on the road to be electric in Europe and China by 2030 [88]. The investment bank Morgan Stanley anticipates only 22% of the distance traveled by car in the USA in 2035 to be realized by electric vehicles, and 50% by 2040, with 25% of new car sales being electric vehicles by 2030 [89], surprisingly low numbers given the current adoption and our model estimates.

In their Global EV Outlook 2023, the International Energy Agency (IEA) projects the worldwide BEC share for 2030 at 15%, according to both of their model scenarios, *Stated Policies Scenario* (STEPS), and *Announced Pledges Scenario* (APS) [90, 91], compared to our result of 31%, following the Bass model, which resembles the slowest adoption within our models. For Europe, the IEA predicts 18%, compared to our 22%, and for the USA, the IEA predicts 16%, compared to our 24%.

Also in contrast to our findings, business intelligence provider Visiongain forecasts that “1 in 12 vehicles sold in California, Germany, South Korea, & Japan, should be hydrogen-powered” [92], and the business analytics platform MarketWatch states their opinion that “Electric vehicles aren’t going to take over any time soon” [93].

Car manufacturers’ positions on BECs differ widely. In 2019, Honda CEO Takahiro Hachigo still claimed that “Electric vehicles will not go mainstream” and stated that Honda will focus on gasoline-electric hybrids, not on battery electric vehicles, through 2030 [94]. Similarly, Toyota Motor Corporation’s former CEO Akio Toyoda has criticized an “excessive hype over electric vehicles” [95]. Toyota will not focus on BECs, but believe that “non-electric cars will also play a lasting role in global auto markets” for the next 30 years, suggesting that “different options including hybrids and fuel-cell vehicles” are needed “to compete against each other” [96]. Contrary to that, a multitude of other carmakers fully embrace BEC technology, with Audi expecting one third of their annual sales to be fully electric by 2025, and Volkswagen aiming for 70% by 2030 [97, 98].

Considering the current state of carbon-neutral powertrain adoption, however, it becomes clear that so far, BEC adoption has by far outperformed all other technologies. For example, in Europe, according to the Eurostat dataset, in 2019, BECs comprised 0.34% of the total passenger car fleet. The second and third-largest fractions of cars with carbon-neutral powertrains, powered by bioethanol and by hydrogen fuel cells, comprised only 0.02% and 0.0003%, respectively.

Thus, with battery electric cars being the only potentially carbon-neutral powertrain option that is currently deployed at large scale, it stands to reason that in the upcoming years, hydrogen fuel cells or the aforementioned related technologies will not be able to catch up and hinder BEC adoption, see also [99]. For instance, even at an estimated 69% compound annual growth rate (CAGR) of the hydrogen fuel cell vehicle market [100], the BEC fleet in Europe in 2029 will still outnumber the fleet of vehicles powered by hydrogen fuel cells by more than three orders of magnitude, based on the data that Eurostat reported for 2019.

In our opinion, even if it is not impossible for hydrogen fuel cells or future technologies to play a significant role eventually—the fact that today those technologies are either niche or non-existent gives the exponentially growing BEC market a significant head start, limiting the influence of competing technologies in the midterm future. Although this argument may not directly apply to regions where the current BEC share is still very small, chances seem high that due to the globalized market, reduction of production costs and sales prices due to mass adoption in other regions will also affect those regions that are still at early stages of BEC adoption, thereby making BECs the most attractive option for customers and manufacturers.

## Conclusions

Battery electric cars are adopted across the globe increasingly rapidly. Our data-driven analysis clearly demonstrates that the current initial phase of worldwide electric car adoption reflects genuine exponential growth. Such fast nonlinear adoption dynamics can be attributed to or even further accelerated by cascades of tipping points, triggered through reinforcing feedbacks, such as policy support, learning-by-doing, economies of scale and the emergence of complementary technologies such as charging infrastructure [101].

Via such diverse factors, the characteristic timescale *a*^−1^ determining the growth rate may change in the future. For instance, slow implementation of charging infrastructure or surging development of alternative emission-free powertrains, based, e.g., on hydrogen, may in principle slow the adoption. In contrast, the current increasing policy support in favor of electric cars, a simpler implementation of autonomous driving for electric vehicles and further commitments of manufacturers or countries to phase out internal combustion engines may accelerate BEC adoption even more.

Extrapolating into the future, we have utilized three different growth models to estimate the point in time at which BECs will dominate the total PC fleet. While the aforementioned factors introduce some uncertainty about the exact time at which the transition to BEC dominance will occur, the exponential nature of the early adoption process makes our predictions robust against slight changes in the growth rate.

Assuming that fundamental disruptions of the BECs adoption process are unlikely, and thus expecting the current trends to continue, we conclude that not only are battery electric cars currently adopted exponentially, they are highly likely to dominate the global passenger car fleet in the near future, less than a decade from today.

## References

[1] IPCC. Synthesis Report (SYR) of the IPCC Sixth Assessment Report (AR6). Intergovernmental Panel on Climate Change; 2023. Available from: https://www.ipcc.ch/report/sixth-assessment-report-cycle/.

[2] Karl TR, Trenberth KE. Modern Global Climate Change. Science. 2003;302(5651):1719–1723. doi: 10.1126/science.1090228 14657489

[3] Höhne N, Blum H, Fuglestvedt J, Skeie RB, Kurosawa A, Hu G, et al. Contributions of Individual Countries’ Emissions to Climate Change and Their Uncertainty. Clim Change. 2011;106(3):359–391. doi: 10.1007/s10584-010-9930-6

[4] Wei T, Dong W, Yan Q, Chou J, Yang Z, Tian D. Developed and Developing World Contributions to Climate System Change Based on Carbon Dioxide, Methane and Nitrous Oxide Emissions. Adv Atmos Sci. 2016;33(5):632–643. doi: 10.1007/s00376-015-5141-4

[5] Chapman L. Transport and Climate Change: A Review. J Transp Geogr. 2007;15(5):354–367. doi: 10.1016/j.jtrangeo.2006.11.008

[6] Sacchi R, Bauer C, Cox B, Mutel C. When, Where and How Can the Electrification of Passenger Cars Reduce Greenhouse Gas Emissions? Renew Sustain Energy Rev. 2022;162:112475. doi: 10.1016/j.rser.2022.112475

[7] Wuebbles DJ, Jain AK. Concerns about Climate Change and the Role of Fossil Fuel Use. Fuel Process Technol. 2001;71(1):99–119. doi: 10.1016/S0378-3820(01)00139-4

[8] Ritchie H, Roser M, Rosado P. CO_2_ and Greenhouse Gas Emissions; 2020. Available from: https://ourworldindata.org/co2-and-greenhouse-gas-emissions.

[9] Solaymani S. CO2 Emissions Patterns in 7 Top Carbon Emitter Economies: The Case of Transport Sector. Energy (Oxf). 2019;168:989–1001. doi: 10.1016/j.energy.2018.11.145

[10] Nikolaidis P, Poullikkas A. A Comparative Overview of Hydrogen Production Processes. Renew Sustain Energy Rev. 2017;67:597–611. doi: 10.1016/j.rser.2016.09.044

[11] da Silva Veras T, Mozer TS, da Costa Rubim Messeder dos Santos D, da Silva César A. Hydrogen: Trends, Production and Characterization of the Main Process Worldwide. Int J Hydrogen Energy. 2017;42(4):2018–2033. doi: 10.1016/j.ijhydene.2016.08.219

[12] Wilberforce T, El-Hassan Z, Khatib FN, Al Makky A, Baroutaji A, Carton JG, et al. Developments of Electric Cars and Fuel Cell Hydrogen Electric Cars. Int J Hydrogen Energy. 2017;42(40):25695–25734. doi: 10.1016/j.ijhydene.2017.07.054

[13] Miao Y, Hynan P, von Jouanne A, Yokochi A. Current Li-Ion Battery Technologies in Electric Vehicles and Opportunities for Advancements. Energies (Basel). 2019;12(6):1074. doi: 10.3390/en12061074

[14] Boretti A. Hydrogen Internal Combustion Engines to 2030. Int J Hydrogen Energy. 2020;45(43):23692–23703. doi: 10.1016/j.ijhydene.2020.06.022

[15] Ababneh H, Hameed BH. Electrofuels as Emerging New Green Alternative Fuel: A Review of Recent Literature. Energy Convers Manag. 2022;254:115213. doi: 10.1016/j.enconman.2022.115213

[16] Brynolf S, Taljegard M, Grahn M, Hansson J. Electrofuels for the Transport Sector: A Review of Production Costs. Renew Sustain Energy Rev. 2018;81:1887–1905. doi: 10.1016/j.rser.2017.05.288

[17] Tatin A, Bonin J, Robert M. A Case for Electrofuels. ACS Energy Lett. 2016;1(5):1062–1064. doi: 10.1021/acsenergylett.6b00510

[18] Lester MS, Bramstoft R, Münster M. Analysis on Electrofuels in Future Energy Systems: A 2050 Case Study. Energy (Oxf). 2020;199:117408. doi: 10.1016/j.energy.2020.117408

[19] Vaalma C, Buchholz D, Weil M, Passerini S. A Cost and Resource Analysis of Sodium-Ion Batteries. Nat Rev Mater. 2018;3(4):1–11. doi: 10.1038/natrevmats.2018.13

[20] Fichtner M. Recent Research and Progress in Batteries for Electric Vehicles. Batter Supercaps. 2022;5(2):e202100224. doi: 10.1002/batt.202100224

[21] Korberg AD, Thellufsen JZ, Skov IR, Chang M, Paardekooper S, Lund H, et al. On the Feasibility of Direct Hydrogen Utilisation in a Fossil-Free Europe. Int J Hydrogen Energy. 2023;48(8):2877–2891. doi: 10.1016/j.ijhydene.2022.10.170

[22] Kalghatgi G. Is It Really the End of Internal Combustion Engines and Petroleum in Transport? Appl Energy. 2018;225:965–974. doi: 10.1016/j.apenergy.2018.05.076

[23] Mpoi G, Milioti C, Mitropoulos L. Factors and Incentives That Affect Electric Vehicle Adoption in Greece. International Journal of Transportation Science and Technology. 2023. doi: 10.1016/j.ijtst.2023.01.002

[24] Lioutas V, Adamos G, Nathanail E. How Ready Are Greek Consumers to Use Electric Vehicles? In: Nathanail EG, Adamos G, Karakikes I, editors. Adv. Mobil.—Serv. Syst. Advances in Intelligent Systems and Computing. Cham: Springer International Publishing; 2021. p. 760–769.

[25] Brückmann G, Willibald F, Blanco V. Battery Electric Vehicle Adoption in Regions without Strong Policies. Transportation Research Part D: Transport and Environment. 2021;90:102615. doi: 10.1016/j.trd.2020.102615

[26] Bjerkan KY, Nørbech TE, Nordtømme ME. Incentives for Promoting Battery Electric Vehicle (BEV) Adoption in Norway. Transportation Research Part D: Transport and Environment. 2016;43:169–180. doi: 10.1016/j.trd.2015.12.002

[27] Singh V, Singh V, Vaibhav S. Analysis of Electric Vehicle Trends, Development and Policies in India. Case Studies on Transport Policy. 2021;9(3):1180–1197. doi: 10.1016/j.cstp.2021.06.006

[28] Berkeley N, Jarvis D, Jones A. Analysing the Take up of Battery Electric Vehicles: An Investigation of Barriers amongst Drivers in the UK. Transportation Research Part D: Transport and Environment. 2018;63:466–481. doi: 10.1016/j.trd.2018.06.016

[29] Wicki M, Brückmann G, Bernauer T. How to Accelerate the Uptake of Electric Cars? Insights from a Choice Experiment. Journal of Cleaner Production. 2022;355:131774. doi: 10.1016/j.jclepro.2022.131774

[30] Tran M, Banister D, Bishop JDK, McCulloch MD. Realizing the Electric-Vehicle Revolution. Nature Clim Change. 2012;2(5):328–333. doi: 10.1038/nclimate1429

[31] Morton C, Anable J, Yeboah G, Cottrill C. The Spatial Pattern of Demand in the Early Market for Electric Vehicles: Evidence from the United Kingdom. Journal of Transport Geography. 2018;72:119–130. doi: 10.1016/j.jtrangeo.2018.08.020

[32] Selena Sheng M, Wen L, Sharp B, Du B, Ranjitkar P, Wilson D. A Spatio-Temporal Approach to Electric Vehicle Uptake: Evidence from New Zealand. Transportation Research Part D: Transport and Environment. 2022;105:103256. doi: 10.1016/j.trd.2022.103256

[33] Rezvani Z, Jansson J, Bodin J. Advances in Consumer Electric Vehicle Adoption Research: A Review and Research Agenda. Transportation Research Part D: Transport and Environment. 2015;34:122–136. doi: 10.1016/j.trd.2014.10.010

[34] Kumar RR, Alok K. Adoption of Electric Vehicle: A Literature Review and Prospects for Sustainability. Journal of Cleaner Production. 2020;253:119911. doi: 10.1016/j.jclepro.2019.119911

[35] Austmann LM. Drivers of the Electric Vehicle Market: A Systematic Literature Review of Empirical Studies. Finance Research Letters. 2021;41:101846. doi: 10.1016/j.frl.2020.101846

[36] Bhat FA, Verma A. A Bibliometric Analysis and Review of Adoption Behaviour of Electric Vehicles. Transp in Dev Econ. 2022;9(1):5. doi: 10.1007/s40890-022-00175-2

[37] Statharas S, Moysoglou Y, Siskos P, Zazias G, Capros P. Factors Influencing Electric Vehicle Penetration in the EU by 2030: A Model-Based Policy Assessment. Energies (Basel). 2019;12(14):2739. doi: 10.3390/en12142739

[38] Kapustin NO, Grushevenko DA. Long-Term Electric Vehicles Outlook and Their Potential Impact on Electric Grid. Energy Policy. 2020;137:111103. doi: 10.1016/j.enpol.2019.111103

[39] Zhang R, Hanaoka T. Deployment of Electric Vehicles in China to Meet the Carbon Neutral Target by 2060: Provincial Disparities in Energy Systems, CO2 Emissions, and Cost Effectiveness. Resources, Conservation and Recycling. 2021;170:105622. doi: 10.1016/j.resconrec.2021.105622

[40] Rietmann N, Hügler B, Lieven T. Forecasting the Trajectory of Electric Vehicle Sales and the Consequences for Worldwide CO2 Emissions. J Clean Prod. 2020;261:121038. doi: 10.1016/j.jclepro.2020.121038

[41] OICA. Vehicles in Use;. Available from: https://www.oica.net/category/vehicles-in-use/.

[42] IEA. Global EV Outlook 2022—Securing Supplies for an Electric Future. International Energy Agency; 2022. Available from: https://www.iea.org/reports/global-ev-outlook-2022.

[43] eurostat. Passenger Cars, by Type of Motor Energy; 2023. Available from: https://ec.europa.eu/eurostat/databrowser/view/ROAD_EQS_CARPDA__custom_2967363/default/table?lang=en.

[44] European Union. Glossary for Transport Statistics. European Union; 2019. 5. Available from: https://ec.europa.eu/eurostat/web/products-manuals-and-guidelines/-/ks-gq-19-004.

[45] Federal Highway Administration. Highway Statistics Series; 2022. Available from: https://www.fhwa.dot.gov/policyinformation/statistics.cfm.

[46] Statistics Iceland. Registered Motor Vehicles 1950-2021; 2022. Available from: https://statice.is/statistics/environment/transport/vehicles/.

[47] Botelho A, Pinto LC. The Diffusion of Cellular Phones in Portugal. Telecommunications Policy. 2004;28(5):427–437. doi: 10.1016/j.telpol.2003.11.006

[48] Katsamaki A, Skiadas CH. Analytic Solution and Estimation of Parameters on a Stochastic Exponential Model for a Technological Diffusion Process. Appl Stoch Models Data Anal. 1995;11(1):59–75. doi: 10.1002/asm.3150110108

[49] Wind Y, Robertson TS, Fraser C. Industrial Product Diffusion by Market Segment. Industrial Marketing Management. 1982;11(1):1–8. doi: 10.1016/0019-8501(82)90028-1

[50] Skiadas C. Two Generalized Rational Models for Forecasting Innovation Diffusion. Technological Forecasting and Social Change. 1985;27(1):39–61. doi: 10.1016/0040-1625(85)90003-4

[51] Griliches Z. Hybrid Corn: An Exploration in the Economics of Technological Change. Econometrica. 1957;25(4):501–522. doi: 10.2307/1905380

[52] Frank LD. An Analysis of the Effect of the Economic Situation on Modeling and Forecasting the Diffusion of Wireless Communications in Finland. Technol Forecast Soc Change. 2004;71(4):391–403. doi: 10.1016/S0040-1625(02)00392-X

[53] Gruber H, Verboven F. The Diffusion of Mobile Telecommunications Services in the European Union. Eur Econ Rev. 2001;45(3):577–588. doi: 10.1016/S0014-2921(00)00068-4

[54] Lee M, Cho Y. The Diffusion of Mobile Telecommunications Services in Korea. Appl Econ Lett. 2007;14(7):477–481. doi: 10.1080/13504850500461431

[55] Liikanen J, Stoneman P, Toivanen O. Intergenerational Effects in the Diffusion of New Technology: The Case of Mobile Phones. Int J Ind Organ. 2004;22(8):1137–1154. doi: 10.1016/j.ijindorg.2004.05.006

[56] Bass FM. New Product Growth Model for Consumer Durables. Manage Sci. 1969;15(5):215–227. doi: 10.1287/mnsc.15.5.215

[57] Qian L, Soopramanien D. Using Diffusion Models to Forecast Market Size in Emerging Markets with Applications to the Chinese Car Market. J Bus Res. 2014;67(6):1226–1232. doi: 10.1016/j.jbusres.2013.04.008

[58] Sundqvist S, Frank L, Puumalainen K. The Effects of Country Characteristics, Cultural Similarity and Adoption Timing on the Diffusion of Wireless Communications. J Bus Res. 2005;58(1):107–110. doi: 10.1016/S0148-2963(02)00480-0

[59] Wu FS, Chu WL. Diffusion Models of Mobile Telephony. J Bus Res. 2010;63(5):497–501. doi: 10.1016/j.jbusres.2009.04.008

[60] Bottomley PA, Fildes R. The Role of Prices in Models of Innovation Diffusion. J Forecast. 1998;17(7):539–555. doi: 10.1002/(SICI)1099-131X(199812)17:7<539::AID-FOR684>3.0.CO;2-S

[61] Dekimpe MG, Parker PM, Sarvary M. Staged Estimation of International Diffusion Models: An Application to Global Cellular Telephone Adoption. Technol Forecast Soc Change. 1998;57(1):105–132. doi: 10.1016/S0040-1625(97)00085-1

[62] Jukić D. Total Least Squares Fitting Bass Diffusion Model. Math Comput Model. 2011;53(9):1756–1770. doi: 10.1016/j.mcm.2010.12.054

[63] GoodCarBadCar. Belgium Car Sales Data;. Available from: https://www.goodcarbadcar.net/belgium-car-sales-data/.

[64] GoodCarBadCar. Denmark Car Sales Data;. Available from: https://www.goodcarbadcar.net/denmark-car-sales-data/.

[65] GoodCarBadCar. Finland Car Sales Data;. Available from: https://www.goodcarbadcar.net/finland-car-sales-data/.

[66] GoodCarBadCar. France Car Sales Data;. Available from: https://www.goodcarbadcar.net/france-car-sales-data/.

[67] GoodCarBadCar. Greece Car Sales Data;. Available from: https://www.goodcarbadcar.net/greece-car-sales-data/.

[68] GoodCarBadCar. Netherlands Car Sales Data;. Available from: https://www.goodcarbadcar.net/netherlands-car-sales-data/.

[69] GoodCarBadCar. Germany Car Sales Data;. Available from: https://www.goodcarbadcar.net/germany-car-sales-data/.

[70] GoodCarBadCar. Poland Car Sales Data;. Available from: https://www.goodcarbadcar.net/poland-car-sales-data/.

[71] GoodCarBadCar. Spain Car Sales Data;. Available from: https://www.goodcarbadcar.net/spain-car-sales-data/.

[72] GoodCarBadCar. Portugal Car Sales Data;. Available from: https://www.goodcarbadcar.net/portugal-car-sales-data/.

[73] GoodCarBadCar. Iceland Car Sales Data;. Available from: https://www.goodcarbadcar.net/iceland-car-sales-data/.

[74] GoodCarBadCar. Italy Car Sales Data;. Available from: https://www.goodcarbadcar.net/italy-car-sales-data/.

[75] GoodCarBadCar. Norway Car Sales Data;. Available from: https://www.goodcarbadcar.net/norway-car-sales-data/.

[76] GoodCarBadCar. Sweden Car Sales Data;. Available from: https://www.goodcarbadcar.net/sweden-car-sales-data/.

[77] GoodCarBadCar. Switzerland Car Sales Data;. Available from: https://www.goodcarbadcar.net/switzerland-car-sales-data/.

[78] GoodCarBadCar. United Kingdom Auto Industry Sales Figures – Total New Vehicle Sales Since 2000;. Available from: https://www.goodcarbadcar.net/uk-total-auto-industry-sales-figures/.

[79] GoodCarBadCar. U.S. Auto Industry Sales Figures—Total New Vehicle Sales Since 1970;. Available from: https://www.goodcarbadcar.net/usa-auto-industry-total-sales-figures/.

[80] CEIC Data. US Motor Vehicle Sales: Passenger Cars, 2005–2023;. Available from: https://www.ceicdata.com/en/indicator/united-states/motor-vehicle-sales-passenger-cars.

[81] Wappelhorst S. Update on Government Targets for Phasing out New Sales of Internal Combustion Engine Passenger Cars; 2021. Available from: https://theicct.org/publication/update-on-government-targets-for-phasing-out-new-sales-of-internal-combustion-engine-passenger-cars/.

[82] European Commission. Zero Emission Vehicles: First ‘Fit for 55’ Deal Will End the Sale of New CO2 Emitting Cars in Europe by 2035; 2022. Available from: https://ec.europa.eu/commission/presscorner/detail/en/ip_22_6462.

[83] International Council on Clean Transportation. Zero-Emission Vehicle Phase-Ins; 2023. Available from: https://theicct.org/zev-phase-ins/.

[84] Brown AL, Fleming KL, Safford HR. Prospects for a Highly Electric Road Transportation Sector in the USA. Curr Sustainable Renewable Energy Rep. 2020;7(3):84–93. doi: 10.1007/s40518-020-00155-3

[85] Kah M. Electric Vehicle Penetration and Its Impact on Glob-al Oil Demand: A Survey of 2019 Forecast Trends; 2019. Available from: https://www.energypolicy.columbia.edu/research/report/electric-vehicle-penetration-and-its-impact-global-oil-demand-survey-2019-forecast-trends.

[86] RethinkX. Rethinking Transportation 2020-2030. RethinkX; 2017. Available from: https://www.rethinkx.com/transportation-report.

[87] Kaas HW, Mohr D, Gao P, Müller N, Wee D, Hensley R, et al. Automotive Revolution—Perspective towards 2030: How the Convergence of Disruptive Technology-Driven Trends Could Transform the Auto Industry. McKinsey & Company; 2016. Available from: https://www.mckinsey.com/industries/automotive-and-assembly/our-insights/disruptive-trends-that-will-transform-the-auto-industry/de-de.

[88] Sylvia T. The Future of Cars Is Electric—but How Soon Is This Future?; 2020. Available from: https://pv-magazine-usa.com/2020/05/19/the-future-of-cars-is-electric-but-how-soon-is-this-future/.

[89] Elkins M. Morgan Stanley Remains Bullish as Tesla (TSLA) Prepares to Welcome Ford and GM to Charging Network; June 15, 2023 8:42 AM EDT. Available from: https://www.streetinsider.com/Corporate+News/-/21802930.html.

[90] IEA. Global EV Outlook 2023: Catching up with Climate Ambitions. International Energy Agency; 2023. Available from: https://www.iea.org/reports/global-ev-outlook-2023.

[91] IEA. Global EV Outlook 2023—Data Product; 2023. Available from: https://www.iea.org/data-and-statistics/data-product/global-ev-outlook-2023.

[92] visiongain. Fuel Cell Electric Vehicle (FCEV) Market Report 2022-2032. Visiongain; 2022. Available from: https://www.visiongain.com/report/fcev-market-2022/.

[93] Brandus P. Opinion: Electric Vehicles Aren’t Going to Take over Any Time Soon; 2021. Available from: https://www.marketwatch.com/story/electric-vehicles-arent-going-to-take-over-any-time-soon-11609882954.

[94] Greimel H, Butters J. Hachigo: Honda Stays Realistic on EVs, AVs; 2019. Available from: https://www.autonews.com/mobility-report/hachigo-honda-stays-realistic-evs-avs.

[95] Landers P. Toyota’s Chief Says Electric Vehicles Are Overhyped. Wall St J (East Ed). 2020;.

[96] Davis R, Inajima T. It’s Too Early to Focus on Electric Cars Only, Toyota Says. Bloombergcom. 2021;.

[97] Poliscanova J. German Carmakers Bet Future on Electric; 2021. Available from: https://www.transportenvironment.org/news/german-carmakers-bet-future-electric.

[98] Allgemeiner Deutscher Automobil-Club e.V. Das Aus für den Verbrenner: Wann wird welcher Hersteller elektrisch?; 2021. Available from: https://www.adac.de/rund-ums-fahrzeug/elektromobilitaet/kaufen/neue-elektroautos/.

[99] Albatayneh A, Juaidi A, Jaradat M, Manzano-Agugliaro F. Future of Electric and Hydrogen Cars and Trucks: An Overview. Energies (Basel). 2023;16(7):3230. doi: 10.3390/en16073230

[100] Market Research Future. Global Hydrogen Fuel Cell Vehicle Market Research Report—Forecast to 2030. Market Research Future; 2023. Available from: https://www.marketresearchfuture.com/reports/hydrogen-fuel-cell-vehicle-market-4722.

[101] Sharpe S, Lenton TM. Upward-Scaling Tipping Cascades to Meet Climate Goals: Plausible Grounds for Hope. Clim Policy. 2021;21(4):421–433. doi: 10.1080/14693062.2020.1870097

